# Selected Ionotropic Receptors and Voltage-Gated Ion Channels: More Functional Competence for Human Induced Pluripotent Stem Cell (iPSC)-Derived Nociceptors

**DOI:** 10.3390/brainsci10060344

**Published:** 2020-06-03

**Authors:** Clemens L. Schoepf, Maximilian Zeidler, Lisa Spiecker, Georg Kern, Judith Lechner, Kai K. Kummer, Michaela Kress

**Affiliations:** Institute of Physiology, Medical University of Innsbruck, 6020 Innsbruck, Austria; clemens.schoepf@i-med.ac.at (C.L.S.); maximilian.zeidler@student.i-med.ac.at (M.Z.); lisa.spiecker@uni-oldenburg.de (L.S.); georg.kern@i-med.ac.at (G.K.); judith.lechner@i-med.ac.at (J.L.); kai.kummer@i-med.ac.at (K.K.K.)

**Keywords:** TRPV1, HCN channel, voltage-gated calcium channel, KCC3, synaptic varicosity, p75, sensory neuron, pain, analgesic

## Abstract

Preclinical research using different rodent model systems has largely contributed to the scientific progress in the pain field, however, it suffers from interspecies differences, limited access to human models, and ethical concerns. Human induced pluripotent stem cells (iPSCs) offer major advantages over animal models, i.e., they retain the genome of the donor (patient), and thus allow donor-specific and cell-type specific research. Consequently, human iPSC-derived nociceptors (iDNs) offer intriguingly new possibilities for patient-specific, animal-free research. In the present study, we characterized iDNs based on the expression of well described nociceptive markers and ion channels, and we conducted a side-by-side comparison of iDNs with mouse sensory neurons. Specifically, immunofluorescence (IF) analyses with selected markers including early somatosensory transcription factors (BRN3A/ISL1/RUNX1), the low-affinity nerve growth factor receptor (p75), hyperpolarization-activated cyclic nucleotide-gated channels (HCN), as well as high voltage-gated calcium channels (VGCC) of the Ca_V_2 type, calcium permeable TRPV1 channels, and ionotropic GABA_A_ receptors, were used to address the characteristics of the iDN phenotype. We further combined IF analyses with microfluorimetric Ca^2+^ measurements to address the functionality of these ion channels in iDNs. Thus, we provide a detailed morphological and functional characterization of iDNs, thereby, underpinning their enormous potential as an animal-free alternative for human specific research in the pain field for unveiling pathophysiological mechanisms and for unbiased, disease-specific personalized drug development.

## 1. Introduction

Over the past decades, animal experiments have been the gold standard for pain research and, without question, have tremendously contributed to the current knowledge in the field. However, besides ethical concerns, the results achieved in rodent models are confounded by interspecies differences when translating findings to humans. Translational approaches using human tissues have become increasingly important, however, are limited by ethical concerns and the accessibility of human life tissue. The dorsal root ganglia (DRG) and the trigeminal ganglia, which contain the majority of primary sensory neurons, are unattainable in a living human being and have even proven complicated to be obtained post mortem [1]. The caveat of species-to-species differences has become evident by recent studies that have compared specific nociceptive markers between human post-mortem and mouse neurons obtained from the DRG; the human proteome includes 286 proteins that are not even expressed in rodent DRG [2]. Functional neuron-like cells differentiated from human induced pluripotent stem cells (iPSCs) provide an elegant way to circumvent interspecies differences, and a protocol to robustly generate human iPSCs-derived nociceptors (iDNs) with low inter cell line difference has become available [3,4]. In patch-clamp experiments, iDNs express tetrodotoxin-sensitive (TTXs) and -resistant (TTXr) voltage-gated sodium currents with unexpected differences to their counterparts from rodent DRG. Novel mechanistic insight into the development of rare congenital disorders with an inability to feel pain or extreme pain has emerged from patient-derived nociceptor studies [5,6,7,8,9,10,11,12]. Even transdifferentiation from fibroblasts has successfully generated neuron-like cells, which express the multimodal transducer ion channel TRPV1 and can be used to study pain mechanisms [13]. However, although such differentiation protocols are well established, functional analyses of iDNs as a quality control have not sufficiently been provided to date. Despite the intriguing potential of patient-derived iDNs, surprisingly, little information is available to date regarding the functional expression of relevant other ion channels and receptors such as hyperpolarization-activated and cyclic nucleotide-gated pacemaker channels (HCN), setting nociceptor excitability and ongoing discharge after injury [14] of voltage-gated Ca^2+^ channels or ionotropic GABA_A_ receptors, which are relevant for synaptic transmission in the superficial dorsal horn [15,16,17,18] and the existence of possible synaptic connections between iDNs potentially forming neuronal networks in a dish.

To start filling this gap, we used a modified protocol for optimized human iDN differentiation [4] and recorded evoked Ca^2+^ transients at different time points during differentiation (D40 and D60) to obtain novel insights into the presence and function of relevant ion channels in iDNs with a specific focus on the high voltage-gated calcium channels of the Ca_V_2-type (Ca_V_2.1–2.3), HCN channels (HCN1–4), as well as ionotropic GABA_A_ receptors and directly compared human iDNs with mouse primary sensory neuron cultures.

## 2. Materials and Methods

### 2.1. Differentiation of Human iPSCs into Nociceptors by Small Molecule Inhibition

The human iPSC line had been generated within the StemBANCC project (part of an Innovative Medicines Initiative) based on a commercially available fibroblast line (Lonza, Basel, Switzerland) sourced from a healthy male donor [19]. The cells were routinely cultured on cell culture plastic ware (Greiner, Kremsmünster, Austria) coated with a murine ECM preparation (Matrigel™ at 8 µg/cm^2^, Corning). Cells were fed daily with mTeSR1 medium (STEMCELL Technologies) and passaged twice a week by calcium withdrawal (0.02% Ethylenediaminetetraacetic acid (EDTA) in phosphate-buffered saline (PBS). The cells were used from the earliest available passage number (P24) up to P40 maximum.

**Figure 1:**
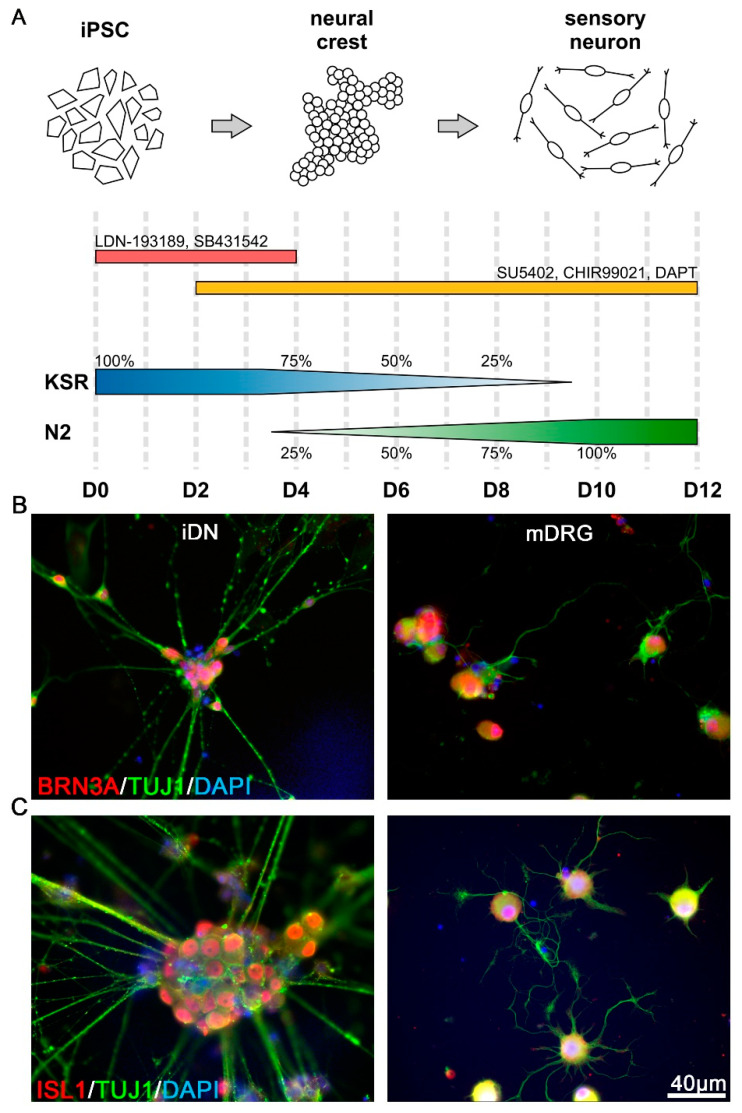
Differentiation of human induced pluripotent stem cells (iPSCs) into nociceptors. (**A**) Schematic representation of the human iPSC differentiation protocol into human iPSC-derived nociceptors (iDNs). Dual SMAD inhibition by LDN193189 and SB431542 was used to induce the neuralization process in knockout serum replacement (KSR) media. Nociceptor induction was further promoted by inhibition of GSK3β, VEGF, and Notch signaling by SU5402, CHIR99021, and DAPT. From D12 on, iDNs were passaged and cultured for several weeks in Neurobasal Medium supplemented with 25 ng/mL NGF, BDNF, and GDNF; (**B**) Both iDNs and mouse dorsal root ganglion neurons (mDRG) expressed the transcription factor BRN3a. Individual iDNs robustly expressed BRN3A in the soma, similar to adult mouse neurons, 100% BRN3A+ cells were detected based on DAPI counterstaining, respectively; (**C**) ISL1 was strongly expressed both in iDNs and mouse neurons, typical somatic clustering of iDNs occurred with aggregates of 10–40 neurons, and 100% ISL1+ cells were detected based on DAPI counterstaining, respectively. Cells from 2–3 individual differentiations were used, scale bar 40 µm.

Differentiation of human iPSCs to iDNs was performed by dual SMAD inhibition, leading to neural crest-like cells followed by an overlapping inhibition of glycogen synthase kinase 3 (GSK3β), vascular endothelial growth factor (VEGF), and Notch signaling (3i inhibition), and finally long-term maintenance in neurobasal medium infused with the neurotrophic factors glial cell line-derived neurotrophic factor (GDNF), nerve growth factor (NGF), and brain-derived neurotrophic factor (BDNF), as firstly described by Chambers, Qi, Mica, Lee, Zhang, Niu, Bilsland, Cao, Stevens, Whiting, Shi and Studer [4]. This protocol was adapted by using 8 days rather than 6 days of 3i inhibition and N2/B27 Neurobasal medium supplemented with NGF, BDNF, and GDNF (Thermo Fischer, Waltham, MA, USA) omitting NT3 for maturation of iDNs (Figure 1A). Briefly, a single cell solution of human iPSCs was prepared using TrypLE Express and 8 × 10^4^ cells were seeded into Matrigel-coated 6-well plates and maintained in mTeSR1 medium. For this and all the subsequent passages, 10 µM of Rho-associated coiled-coil containing protein kinase (ROCK) inhibitor (Y27632, Sigma-Aldrich, St. Louis, MO, USA) was added at the time of the passage, for 1 day, to increase cell survival. The human iPSC cells were maintained in mTeSR1 medium until they reached 60–70% confluency before differentiation was initiated.

Subsequently, dual SMAD inhibition was initiated, thereby suppressing TGF-beta and BMP4, in knockout serum replacement (KSR) medium Knockout DMEM (Gibco), 15% KO serum replacement (Gibco), 1% non-essential amino acids (PAA), 2 mM Glutamax (Gibco), 1x penicillin/streptomycin (Sigma-Aldrich), and 100 µM β-mercaptoethanol (Gibco) supplemented with 100 nM of LDN-1931189 (Stemgent, ALK2/3 inhibitor, 100 nM, D0–D5), and 10 µM SB431542 (selleckchem.com, ALK5 inhibitor, 10 µM, D0–D5) for 5 days.

Additionally, 3 µM CHIR99021 (selleckchem.com, GSK3β inhibitor, D2–D12), 10 µM DAPT (selleckchem.com, Notch inhibitor, 10 µM, D2–D12), and 10 µM SU5402 (Sigma-Aldrich, VEGF inhibitor; 10 µM, D2–D12) were added from D2–D12. From D4 on, N2/B27 medium (Gibco Neurobasal™ medium, supplemented with 1% N2 (Gibco), 2% B27 without Vitamin A (Gibco), and 2 mM Glutamax (Gibco)), and 1× penicillin/streptomycin (Sigma-Aldrich) was added to the KSR medium and incremented every second day by 25%. For a schematic representation of the exact medium and inhibitor application, see Figure 1A.

On day 12, 30,000 cells were passaged onto Matrigel-coated cell culture imaging dishes (ibidi GmbH, Munich, Germany) or glass coverslips and maintained in N2/B27 medium (exchanged every 2–3 days) supplemented with NGF (hNGF, PeproTech, 25 ng/mL), BDNF (hBDNF, PeproTech, 10 ng/mL), and GDNF (hGDNF, PeproTech, 25 ng/mL), for the remaining time. Alternatively, cells were cryopreserved in liquid nitrogen at D12, so that final maturation could be performed at a later time points.

Cytosine-β-D-arabinofuranoside (4 µM, Sigma-Aldrich) was administered on D14 for ~24 h to reduce the amount of non-differentiated proliferating cells and iDNs were maintained in N2/B27 medium up to 48 days.

### 2.2. Primary Sensory Neuron Culture

Male, 10–12-week-old C57BL/6J mice were used for further experiments. Mice were housed at a constant 12 h light/dark cycle with ad libitum access to water and chow. Lumbar DRG explants were harvested from adult mice and primary sensory cultures prepared as previously published [20]. Briefly, after extraction, the connective tissue was removed and ganglia were incubated in Liberase Blendzyme 1 (collagenase + thermolysin, 9 mg/100 mL DMEM, Roche, Basel, Switzerland) for 2 × 30 min. Subsequently, DRGs were washed and treated with 1× trypsin-EDTA for 15 min, followed by a washing step with TNB™ medium supplemented with L-glutamine (Invitrogen, Waltham, MA, USA), penicillin G sodium, streptomycin sulfate (Invitrogen), and protein-lipid complex (Biochrom, Harvard Bioscience Inc. Holliston, MA, USA). Dissociation of DRG was performed using a fire-polished glass pipette and non-neuronal cells were removed by centrifugation with a 3.5% BSA-gradient. Then, sensory neurons were resuspended in TNB™ medium supplemented with 25 ng/mL NGF and plated on poly-L-lysine/laminin coated coverslips. Cells were used for immunofluorescence microscopy after 23 days of incubation at 37 °C and 5% CO_2_.

### 2.3. Immunofluorescence Microscopy

The AD2 (SbAd-02-01) iPSC-derived iDNs were fixed on D26, D36, D50, and D60 in 4% paraformaldehyde in PBS at room temperature, for 10 min. Fixed iDNs were treated with 5% normal goat serum in PBS supplemented with 0.2% BSA and 0.2% Triton X-100, for 30 min. Primary antibodies were applied at 4 °C overnight, in a humidified chamber, and detected by fluorochrome-conjugated secondary antibodies (Alexa goat anti-rabbit A594 (#A32740), goat anti-mouse A594 (#A32742), goat anti-rabbit A488 (#A32731), and goat anti-mouse A488 (#A32723), 1:4000, Invitrogen). Primary antibodies used were anti-synapsin (1:1000, mouse monoclonal, Synaptic Systems (SySy); #106011 reference [21]); anti-BRN3A (1:500 rabbit polyclonal, SySy, #411003, reference [22]); anti-ISL-1 (1:500, rabbit polyclonal, SySy, #406003 reference [23]); anti-Ca_V_2.1 (1:1000, rabbit polyclonal, SySy, #152103 k.o verified); anti-Ca_V_2.2 (1:1000, rabbit polyclonal, SySy, #152313 reference [24]); anti-Ca_V_2.3 (1:1000, rabbit polyclonal, SySy, #152403 reference [25]); anti-RUNX1 (1:200, rabbit polyclonal, Abcam, #23980 reference [26]); anti-p75 (1:200, rabbit polyclonal, Abcam, #AB52987 reference [27]); anti-NKCC3 (1:500, rabbit polyclonal, Thermo Fisher Scientific #PA5-56975); anti-TRPV1 (1:200, rabbit polyclonal, Alomone Labs, #ACC-030 reference [28]); anti-TUJ1 (1:600, mouse monoclonal, R&D Systems, #MAB1195 reference [29]); anti-GABA_A_R (β2,3 chain, representing the most abundant subunit chains of GABA_A_Rs) (1:1000, mouse monoclonal, Chemicon, #MAB341 reference [21]); and anti-HNC1–4 (1:600, Alomone Labs, HCN1 #APC-056, HCN2 #APC-030, HCN3 #APC-057, and HCN4 #APC-052, reference [30]). Nuclei were counterstained with DAPI (4′,6-diamidino-2-phenylindol) 1:10,000, (Thermo Fisher Scientific). Images were recorded using an Axioimager 2 Microscope (Carl Zeiss Microscopy) with cooled CCD camera (SPOT Imaging Solutions). Average fluorescence intensities were quantified using Metaview software (version 7.8.0.0, Molecular Devices, LLC, San Jose) with the line scan plug-in to quantify fluorescent intensity along a defined line cutting the individual axonal structures.

### 2.4. Microfluorimetric Ca^2+^ Measurements

The Ca^2+^ measurements of iDNs were performed as we previously described [31]. The iDNs were loaded with 3 µM of Fura-2 AM (Invitrogen) in extracellular solution (ECS) containing as follows: 145 mM NaCl, 5 mM KCl, 2 mM CaCl_2_, 1 mM MgCl_2_, 10 mM D-glucose, and 10 mM HEPES with pH adjusted to 7.3 with NaOH, for 30 min, at 37 °C, in 5% CO_2_. Subsequently, cells were washed with ECS. Calcium imaging was performed on an IX71 microscope (Olympus) with 20×/0.85 N.A. oil-immersion objective. The Omicron LEDHUB System was used for excitation of Fura-2 at 340 nm and 380 nm wavelengths, and emission was detected at 510 nm. The F340/F380 excitation ratio was calculated after background correction. The baseline was recorded over 10 s prior to each treatment. To elucidate treatment-induced Ca^2+^ transients in an unbiased way, an increase of 10% over baseline F340/F380 ratio was applied as the threshold. The percentage of active cells was calculated and compared for each treatment and time point.

### 2.5. Statistical Analyses

Statistical analyses were performed in Python (v3.8; https://www.python.org) using the open-source packages “scipy.stats” (https://www.scipy.org), “numpy” (https://www.numpy.org), and “pandas” (https://pandas.pydata.org), as well as GraphPad Prism (v8) (GraphPad Software, San Diego). Data are presented as mean ± standard error of the mean (SEM) or as percentages. Two-way ANOVA with Dunett’s correction for multiple comparisons or Chi-square test without Yates correction was applied as indicated. The significance threshold was set to *p* < 0.05. For graphical illustration, Adobe Photoshop CC 2020 (Adobe San Jose, CA, USA), CorelDraw v8 (Ottawa, ON, Canada), and the Python packages “Seaborn”, “Matplotlib”, and “Pandas” were used.

## 3. Results

### 3.1. Expression of Early Transcription Factors Regulating Sensory Differentiation

Characterization of early stage iDNs and sensory neurons obtained from mature mouse DRG was performed by quantification of BRN3A and ISL1 expression, which are two transcription factors with critical implications for sensory neuron development [32]. In line with previous reports, immature iDNs (D26), as well as mouse sensory neurons, showed a robust somatic expression of both transcription factors (Figure 1B,C) [33]. The iDNs showed a stable somatic expression of BRN3A (Figure 1B), and ISL1 expression was detectable similar to BRN3A in 100% of iDNs depending on the DAPI counterstaining with a threshold of >10 µm as a positive selection criterion (Figure 1C). Furthermore, D26 iDNs showed a characteristic somatic clustering, as described previously [4]; neurites stained positive for the neuron specific β-III tubulin marker TUJ1 and putative axo-axonal synaptic varicosities were visible.

### 3.2. RUNX1 and p75 Expression Reveal a Nociceptor Neuron Phenotype

**Figure 2:**
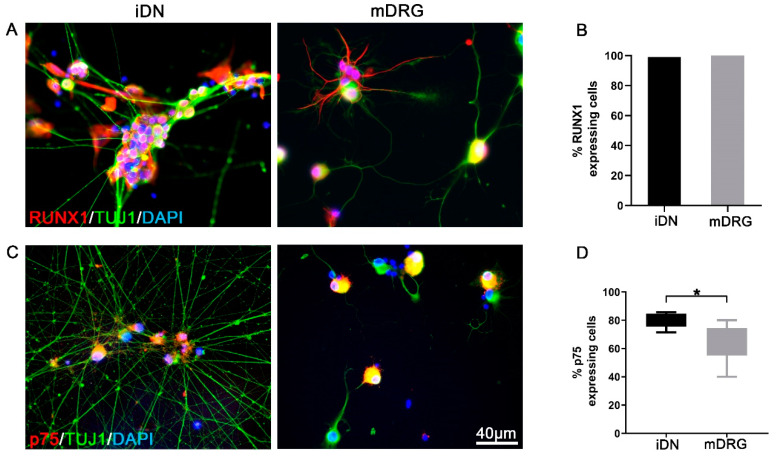
RUNX1 and p75 expression indicative of nociceptor-like phenotype of iDNs. (**A**) Representative image of D36 iDNs as compared with mouse neurons (mDRG); (**B**) Both iDNs and mouse DRGs showed a robust RUNX1 soma expression pattern in 100% of DAPI+ cells. Counting threshold was set to >10 µm based on DAPI counterstaining; (**C**) p75-IR cells in D36 iDNs as compared with mouse neurons; (**D**) ~79% of iDNs were positive for p75, whereas only ~64% were positive in mouse neuron cultures. Cells from 2–3 individual differentiations were used, two-tailed t-test, * *p* = 0.025, error bars indicate SEM, scale bar 40 µm.

Runt-related transcription factors (RUNX) play essential roles during the development of somatosensory neurons. In particular, RUNX1 determines the nociceptor phenotype for pain, itch, and thermal sensation in mature nociceptive neurons [34,35]. RUNX1 together with the T-cell leukemia homeobox 3 protein (TLX3) regulate the development and survival of TrkA expressing nociceptive sensory neurons [36,37], and RUNX1 also plays a pivotal role for the development of low-threshold C-mechanoreceptors (CLTMs) [38]. However, RUNX1 expression persists longer in RET+ neurons during development, but extinguishes in adult TrkA+ neurons [34]. In the current study, we detected stable expression of RUNX1 both in iDNs and mouse neurons (Figure 2A). RUNX1 was expressed in all TUJ1 positive iDNs (Figure 2B). In order to further dissect the phenotype of iDNs, the low affinity nerve growth factor receptor p75 as a broadly accepted nociceptive marker was included in the characterization [39,40,41] and p75 was shown to be required for the sensory neuron diversity by potentiating RET signaling [42], as well as RET was shown to be activated subsequently after RUNX1 expression in previously established iDN differentiation protocols [4]. We detected a robust expression of p75 in iDNs (Figure 2C), and ~79% of iDNs showed p75 abundance as compared with mouse DRGs (~64%) (Figure 2D), and therefore in conjunction with the high expression of RUNX1 resembling a non-peptidergic iDN phenotype (Figure 2C,D). Consequently, the gross majority of differentiated iDNs developed a nociceptive phenotype which resembled well the phenotype of small size sensory neurons obtained from adult mice [34].

### 3.3. TRPV1 Experession in iDNs

**Figure 3:**
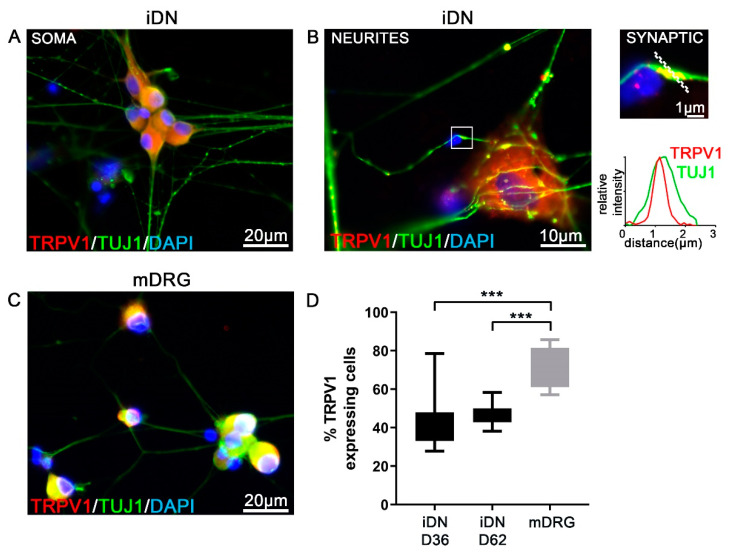
Robust TRPV1 expression in iDNs. (**A**) Robust TRPV1 expression in D36 iDNs, counting threshold was set to >10 µm based on DAPI counterstaining; (**B**) Neurite regions revealed TRPV1 abundance in individual TUJ1-IR varicosities. Relative fluorescence intensity measurements using line scans for individual TUJ1-IR clusters (enlargement) revealed putative synapses including TRPV1 clustering in synaptic regions, line scan was set to 3 µm; (**C**) Mouse DRG neurons (mDRG) were used as controls; (**D**) Quantification of TRPV1+ cells based on the total number of DAPI+ cells in D36 and D62 iDNs. By D36, already ~44% of iDNs showed a strong TRPV1 expression stagnating to ~46% by D62. A significantly higher percentage of ~74% of mouse neurons stained positive for TRPV1. 2–3 individual differentiations were used, one-way ANOVA with Dunett’s multiple comparisons, *** *p* < 0.001, error bars indicate SEM.

**Figure 4:**
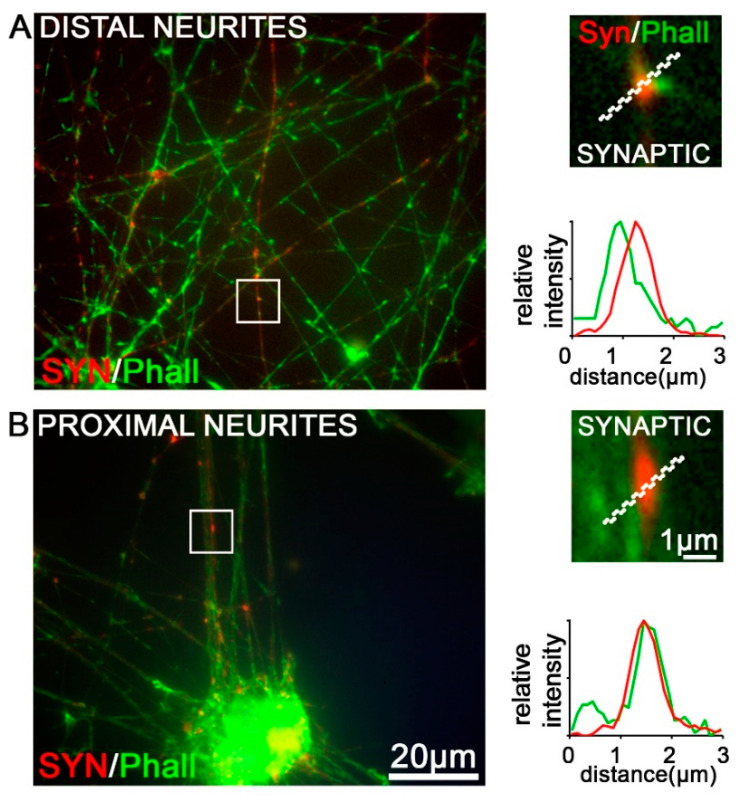
Characterization of synaptic varicosities on D50 iDNs. iDNs were labeled with a synapsin antibody costained for phalloidin 488 (green) indicating F-actin^+^ axonal structures. Magnifications highlight individual synaptic varicosities, putative axo-axonal synapses (red) both in regions of distal (**A**) and proximal neurites (**B)**. 2–3 individual differentiations were used, bar 20 µm, 1 µm (enlargement), line scan was set to 3 µm.

The superfamily of the transient receptor potential (TRP) channels has emerged as the largest group of molecules involved in the transduction of various physical stimuli into neuronal signals in primary sensory neurons [43]. Here, we focused on the noxious heat sensitive channel TRPV1 that can be activated by the vanilloid capsaicin, the main pungent of hot chilli peppers. Stable TRPV1 immunoreactivity (IR) was detected in iDNs and mouse DRGs (Figure 3A–C). By performing triple stainings for TRPV1/TUJ1/DAPI, we identified ~44% of all iDNs to robustly express TRPV1 in the soma at D36, stagnating to ~46% at D62 as compared with ~74% in mouse DRGs (Figure 3D). This result could indicate a mature iDN phenotype at D36. Interestingly, putative TUJ1-IR synaptic varicosities that stained positive for TRPV1 were visible along neurites as quantified by line scan analysis (Figure 3B and Figure 4). To further evaluate these synaptic varicosities, a staining for the presynaptic vesicle-associated protein, synapsin, an early marker for presynaptic vesicle recruitment, was performed [44]. Costaining of synapsin with the toxin phalloidin, an F^+^actin neurite marker, revealed synapsin-positive varicosities in iDNs, thereby, indicating that synaptogenesis, indeed, occurred in these neurons (Figure 4). The majority of the axonal varicosities in iDNs showing phalloidin-IR were also immunoreactive for synapsin both in regions distal and proximal to the next iDN cluster (Figure 4A,B).

### 3.4. Expression of Pacemaker Channel HCN1–3 but Not HCN4 in iDNs and mDRG Neurons

**Figure 5:**
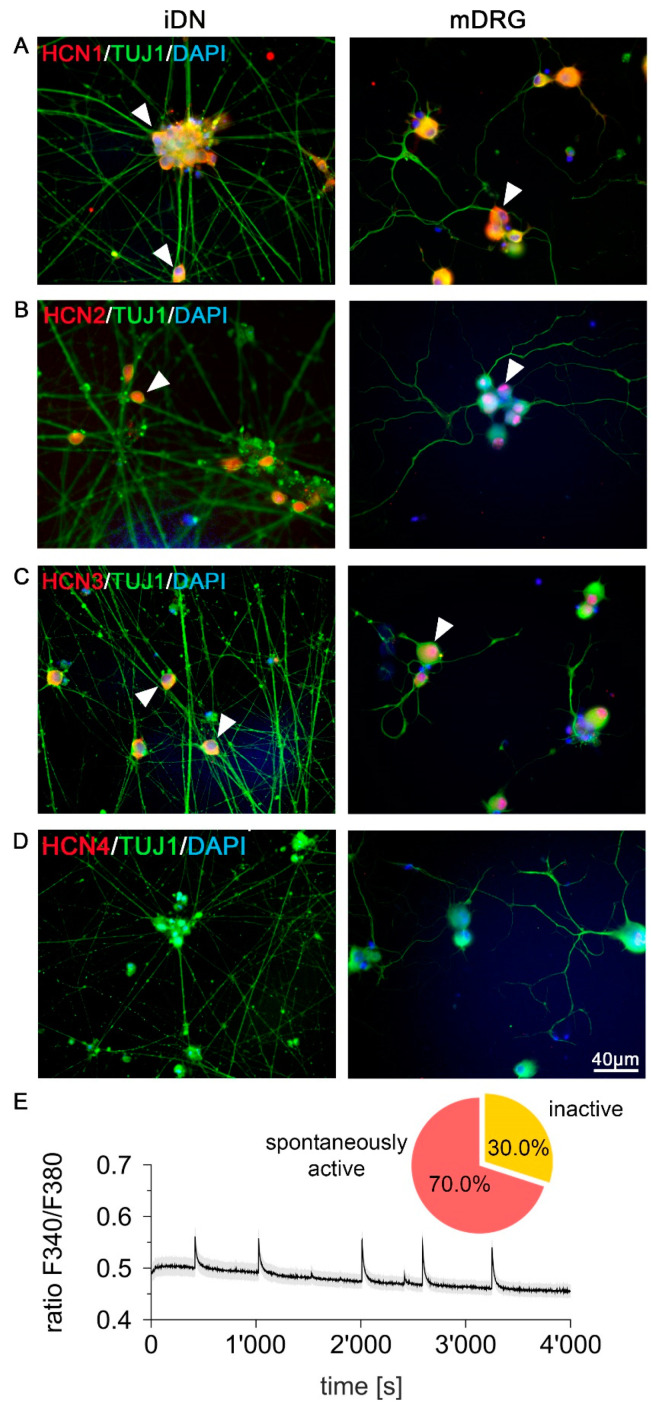
Ongoing activity and expression of pacemaker channels HCN1–3 in iDNs. (**A**–**C**) Immunoreactivity of HCN1–3 was reproducibly detected both in iDNs and mDRG neurons (arrowheads). 100% of cells showed immunoreactivity of HCN1–3 based on DAPI counterstaining, respectively; (**D**) HCN4 was not expressed in D26 iDNs or in mDRG controls, scale bar 40 µm; (**E**) iDNs exhibit a low baseline Ca^2+^ concentration but regular transient increases (ratio F340/F380) indicative of spontaneous discharge activity (*n* = 30). Pie chart indicating the percentage of spontaneously active iDNs (70%) as compared with inactive iDNs (30%) at D60.

Hyperpolarization-activated cyclic nucleotide-gated (HCN) channels drive the repetitive firing in nociceptive neurons, and thus play a crucial role in pace making activity [45]. To further underpin the mature phenotype of iDNs, expression of HCN1–4 was assessed on D26 iDNs and mouse sensory neurons. Immunofluorescent analysis revealed a strong HCN1–3-IR (Figure 5A–C) but not HCN4-IR (Figure 5D) both in D26 iDNs and mouse neurons. In order to explore whether this expression of pacemaker channels was functionally relevant, the spontaneous activity of iDNs was assessed by microfluorometric calcium measurements (Figure 5E). Approximately 70% of iDNs exhibited spontaneous Ca^2+^ transients indicative of spontaneous action potential firing, which was defined by a spontaneous calcium influx, increasing the peak to baseline F340/F380 ratio by a factor of 1.1 in iDNs. Each peak was independently evaluated to a newly calculated baseline preceding the peak 30 s in advance, to compensate for general baseline transient changes (Figure 5E).

### 3.5. Expression of Ca_V_2 High Voltage-Activated Calcium Channels

**Figure 6:**
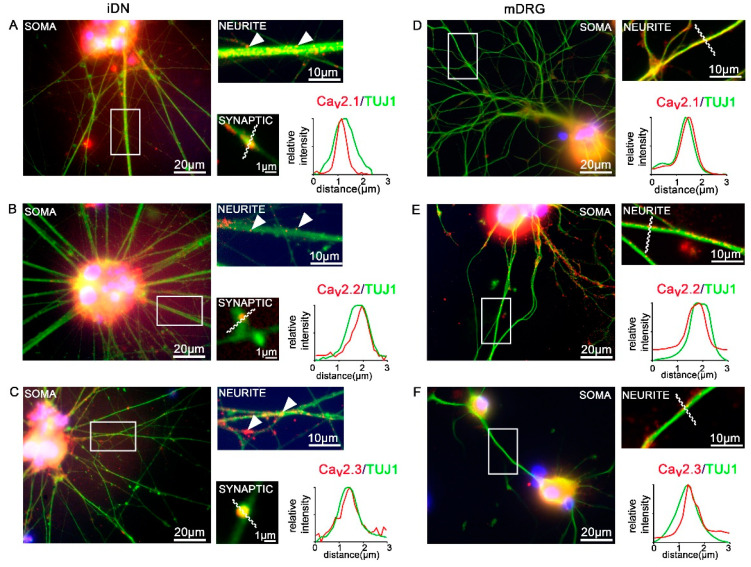
Expression of Ca_V_2 high voltage activated calcium channels. (**A**–**C**) D50 iDNs showed robust expression of all Ca_V_2 high voltage activated calcium channels in regions of the soma, neurites, as well as axonal varicosities. Arrowheads indicate individual (**A**) Ca_V_2.1/PQ-type, (**B**) Ca_V_2.2/N-type, and (**C**) Ca_V_2.3/R-type clusters along neurite structures of iDNs. Line scan measurements for individual Ca_V_2 clusters on TUJ1-IR+ varicosities revealed their location within putative synaptic structures in iDN cultures, line scan was set to 3 µm. (**D**–**F**) Mouse neurons (mDRG) served as controls, line scans performed on neurite segments, no axonal varicosities were detected, 2–3 individual differentiations, scale bars 20 µm (overview, soma), 10 µm (enlargement, axon), 1 µm (axonal varicosities, putative synapses).

Ion channels have proven to be indispensably important for synaptic, and thus, consequently, for nociceptor function. In addition to voltage-gated sodium channels, which have been extensively studied and described as nociceptive ion channels [5,8,9,12,46,47,48,49], here, we focused on high voltage-activated calcium channels of the Ca_V_2.1/PQ-type, Ca_V_2.2/N-type, and Ca_V_2.3/R-type. All three high voltage activated Ca_V_2 channels contribute to nociception [50], and therefore were included in the present comparison between human iDNs (D50) and mouse DRG neurons. We found robust expression of all three Ca_V_2 channels, i.e., Ca_V_2.1/PQ-type, Ca_V_2.2/N-type, and Ca_V_2.3/R-type, which were clustering along neuronal TUJ1+ neurites (Figure 6A–F). In order to address the subcellular location of individual Ca_V_2 channel subtypes, in particular, in putative synaptic varicosities of iDNs, line scan measurements were performed. Relative fluorescence intensity measurements revealed putative synaptic localization patterns of individual Ca_V_2 channel subtypes (red) on TUJ1-IR synaptic varicosities, indicating putative axo-axonal synaptic connections between differentiated iDNs (Figure 6A–C). This was strikingly different in mouse neuron cultures, where stable Ca_V_2 channel expression was found on TUJ-IR neurites, however, no putative axo-axonal synaptic varicosities were observed (Figure 6D–F).

### 3.6. GABAA Receptors and Transporters in iDNs

**Figure 7:**
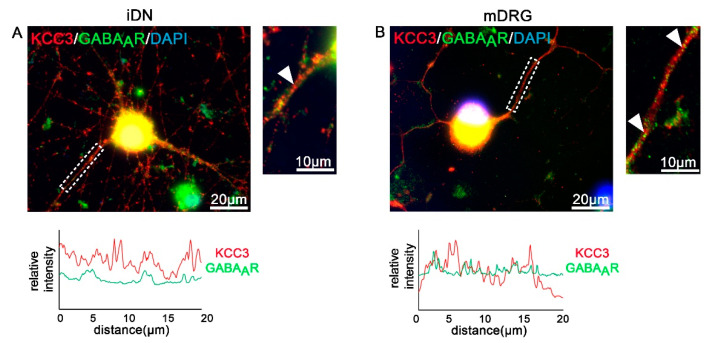
Quantification of KCC3/GABA_A_R clustering on iDNs and mouse DRG neurons. (**A**,**B**) Both day 40 iDNs and mouse DRG neurons stained positive for KCC3-IR and GABA_A_R labeling (arrow heads neurite selections). Line scan analyses on 20 µm neurite structures were performed. (**A**) Lower intensity levels of GABA_A_R clustering in iDNs were elucidated as compared with mouse DRGs (**B**). 2–3 individual differentiations, scale bars 20 µm and 10 µm (enlargement), line scan was set to 20 µm.

In the adult peripheral nervous system, activation of ionotropic GABA_A_ receptors (GABA_A_R) can cause neuron excitation because of the high concentration of intracellular chloride in these neurons (for review see [51]). Potassium-chloride cotransporters (KCCs) appear to play a critical role in hyperalgesia and allodynia following peripheral inflammation or nerve injury [52]. As an example, we investigated localization patterns of KCC3 and GABA_A_R (β-2/3 subunit)-IR in iDNs and mouse sensory neurons. Whereas both proteins were expressed in iDNs and mouse sensory neurons, D40 iDNs showed a different co-clustering pattern as indicated by line scan analysis of neurite segments (Figure 7A,B). This could indicate that D40 iDNs show a lower degree of KCC3/GABA_A_R co-clustering, however, whether this is functionally relevant will be addressed in further experiments.

### 3.7. Functional Characterization of iDNs

**Figure 8:**
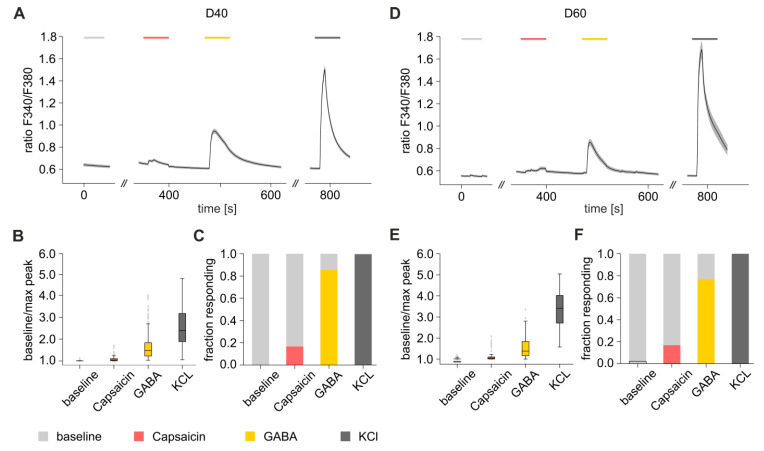
Ca^2+^ transients evoked by chemical nociceptor stimuli in iDNs on D40 (**A**–**C**) and D60 (**D**–**F**). Time-series analysis of substance induced Ca^2+^ transients following administration of extracellular solution (ECS, grey), capsaicin 25 nM (red), GABA 50 µM (yellow) and 25 mM KCl (dark grey) was quantified and visualized. The mean F340/F380 for each positive cell (succeeding the peak-to-baseline threshold of 1.1) was determined and substance induced reaction was compared qualitatively. The stacked bar charts (**C**,**F**) indicate the percentage of positively reacting and non-reacting cells upon administration of each individual substance. Chi-square test did not identify any significant differences between D40 and D60 (*p*-value > 0.05).

In order to further functionally characterize iDNs, microfluorimetric Ca^2+^ measurements of iDNs were performed on D40 (Figure 8A–C) and D60 (Figure 8D–F). For this, cells exhibiting a neuronal morphology were selected for further analysis (D40 189 iDNs and D60 98 iDNs). Averaged time-series analysis of calcium transients of all selected iDNs revealed prominent calcium transients already on D40, after administration of 25 nM capsaicin, 50 µM GABA, and 25 mM KCl (Figure 8A–C). Interestingly, there was a pronounced discrepancy between the percentages of iDNs expressing TRPV1-IR (Figure 3D) and of iDNS responding to the TRPV1 activator capsaicin with fast rising Ca^2+^ transients indicative of Ca^2+^ influx (Figure 8D–F). Additionally, administration of GABA, which activates both GABA_A_ as well as GABA_B_ receptors, induced depolarizations in the majority of iDNs, comparable with those found in rodent DRGs (Figure 8C,F).

However, no significant differences in substance induced Ca^2+^ transients were determined between D40 and D60, indicating a high degree of functional maturation of iDNs regarding the expression of relevant markers and ion channels already by D40, however, full functionality still lagged behind (Figure 8A–F).

Collectively, our study of human iDNs and the direct comparison to mouse sensory neurons of a selected nociceptive marker panel (summarized in Table 1) provides not only morphological evidence for human iDNs developing specific nociceptor phenotypes but, more importantly, provides converging evidence supporting their suitability as a model to study nociceptive mechanisms.

## 4. Discussion

Over the past decades, rodent models have been indispensable for pain research and have largely contributed to our current knowledge about molecular mechanisms of pain [53]. However, the field has sought alternatives due to a limited translation to the human disease situation caused by species-to-species differences [54]. For many years, research on human tissues could only be performed by using post-mortem tissue, which faces restrictions, in particular, as far as functional assays are concerned [1]. An emerging opportunity to circumvent the apparent translational paresis in pain research is the application of new model systems derived from human iPSCs that allow directed cell-type specific programming into iDNs. This approach has become applicable on a larger scale after robust differentiation protocols became available [4,55,56,57]. First attempts to personalize research towards pain disorders arising from gain-of-function mutations of voltage-gated Na^+^ channels have exploited these possibilities [58,59]. More recently, iDN transdifferentiation protocols have speeded up the use of iDNs from patients suffering from monogenetic pain disorders for which, so far, mechanistic insight is unavailable [60]. Specific immunohistochemical markers such as RUNX1, p75, BRN3A, and TRPV1 generally mark the successful transition of human iPSCs towards a nociceptor phenotype [4,34,42,43] and have proven the successful differentiation of iDNs in the current study. However, the functional characteristics of iDNs and their similarities to human nociceptors are still insufficiently explored. In the present study, therefore we provide a detailed characterization of iDNs differentiated from human iPSCs and functional evidence how iDNs partially resemble their rodent counterparts by a comparison with mouse DRG neurons.

In addition to voltage-gated Na^+^ channels, which have been extensively exploited for hereditary painful neuropathies [5,8,9,12,49], all high voltage-activated Ca_V_2 channels have important implications in pain signaling, as reviewed by Bourinet, Altier, Hildebrand, Trang, Salter and Zamponi [50]. The impact of Ca_v_ channels on nociception has been elegantly demonstrated by the application of specific toxins blocking each channel. For example, the Ca_V_2.1 channel blocker agatoxin, a spider toxin, was shown to inhibit inflammatory pain of the knee joints [61]. The Ca_V_2.2/N-type channel blocker conotoxin-GVIA, a sea snail toxin, elicits potent suppression of pain when delivered intrathecally in rodents [62] and the Ca_V_2.3 blocker SNX-482, another spider toxin, additionally elicited analgesia in models of neuropathic pain [63]. In iDNs, we detected a robust expression of all Ca_V_2 high voltage-activated calcium channels at TUJ1-IR axonal varicosities, indicative of synapse formation. These synaptic characterization data are important, considering the central roles of voltage-gated calcium channels and their accessory α_2_δ subunits playing a critical role for neurotransmitter release at presynaptic terminals and further representing direct targets for analgesic drugs including Gabapentinoids [64,65,66]. In contrast, mouse nociceptors showed Ca^2+^ channel expression patterns on axonal segments but no putative synaptic boutons, suggesting that adult nociceptors do not form synaptic networks in culture.

The superfamily of the transient receptor potential (TRP) channels has emerged as the largest family of transducer ion channels in primary sensory neurons [43]. Here, we focused on the nociceptor-specific, heat sensitive TRPV1 channel which was detectable in the somata of ~44% of all iDNs by D36, ~46% by D62, and ~74% of mouse sensory neurons. The discrepancy between the percentages of iDNs expressing TRPV1-IR and of iDNs responding to the TRPV1 activator capsaicin was likely to be caused by TRPV1 sequestration in vesicles by the interaction with t-SNARES of the Vit1b type [67]. Indeed, TRPV1 and the transient receptor potential ankyrin1 (TRPA1) were found in VAMP1 containing vesicles of cultured sensory neurons [68], and thus chemical nociceptor stimulation could not target these fractions.

The iDN processes developed structural signatures resembling synaptic varicosities. Interestingly, Ca_V_2 and also TRPV1 immunoreactivity were found in such synapsin-positive varicosities in iDNs. This finding is in accordance with previous reports that demonstrated a role of TRPV1 in synaptic vesicle recycling [69,70]. The synapses and their key regulatory proteins, in the active zones including Ca_V_2 VGCCs with their auxiliary subunits as part of the synaptic release machinery, are the communication platforms between neurons, and thus critical targets for several analgesics [50,64]. Accordingly, this species-to-species difference could represent an important pathophysiological bias between rodent and human pain model systems and needs to be addressed in full detail.

Our results are also in line with previous reports on HCN1–3 channels that were found in different flavors in small and large diameter rat DRG neurons, with nociceptors expressing, in particular, HCN2, whereas HCN4 levels were negligible [71,72]. These data indicate that, already at D36, iDNs developed a relatively mature nociceptive phenotype or even resembled a suitable model for neuropathic pain, since expression patterns of HCN channels and signatures of ongoing activity, which typically develop after nerve injury, were observed in D26 iDNs [14,73,74,75,76,77,78,79,80,81].

Several functionally relevant receptors which are located in iDNs such as HCN channels, the multimodal transducer channel TRPV1, as well as ionotropic GABA_A_R, are expressed on nociceptor peripheral nerve terminals, slow fiber axons, cell bodies in the DRG, and their central processes connecting to neurons in the spinal dorsal horn [82]. These receptors can serve dual functions. For example, GABA is synthesized in fibroblasts and likely liberated upon injuries causing skin disruption, and GABA_A_ receptor-mediated nociceptor excitation is well accepted [83,84]. In addition, GABA_A_ receptors, at presynaptic terminals in the superficial dorsal horn, can be targeted for presynaptic inhibition, which sets a brake to mechanical hypersensitivity [85]. However, whether GABA has excitatory or inhibitory effects largely depends on the actual concentration of intracellular chloride in these neurons (for review see [51]). Potassium-chloride cotransporters (KCCs) appear to play a critical role in hyperalgesia and allodynia following peripheral inflammation or nerve injury, and typically the KCC3 transcript is expressed at higher levels than the other cation-Cl^-^ cotransporter transcripts in adult DRG neurons [52]. Expression of KCC3, as reported, further suggests that iDNs resembled a relatively mature nociceptor phenotype.

## 5. Conclusions

Altogether, iDNs generated with the modified protocol developed a surprisingly far developed phenotype, which, from D36, combined attributes of nociceptive terminals such as TRPV1 transducer channels with the machinery that is located at presynaptic terminals of primary afferent neurons including all types of high voltage activated Ca_V_2 channels. These findings underpin the enormous potential of these neurons as an animal-free alternative for future personalized pain research, to unveil pathophysiological mechanisms in humans and for unbiased, disease-specific, and patient-oriented drug development.

## Figures and Tables

**Table 1:** Summary of the used markers, level of expression, and morphological abundance in iDNs and mouse sensory neurons.

Marker	iDN	mDRG
**BRN3A**	100% (high and low expressing cells)	100%
**ISL1**	100%	100%
**RUNX1**	100% (high and low expressing cells)	100%
**p75**	~79%	~64%
**TRPV1**	~45%	~74%
**SYN**	positive varicosities/boutons	no varicosities/boutons
**HCN1–3**	100%	100%
**HCN4**	not expressed	not expressed
**Ca_V_2.1** **Ca_V_2.2** **Ca_V_2.3**	expressed, synaptic localization patterns	expressed on neurites, no synaptic localization patterns
**KCC3/GABA_A_R**	expressed/lower levels	expressed/higher levels higher degree of colocalization
**TUJ1/Phall**	neurites and varicosities/boutons	only on neurites
